# TS-GOEA: a web tool for tissue-specific gene set enrichment analysis based on gene ontology

**DOI:** 10.1186/s12859-019-3125-6

**Published:** 2019-11-25

**Authors:** Jiajie Peng, Guilin Lu, Hansheng Xue, Tao Wang, Xuequn Shang

**Affiliations:** 1 School of Computer Science, Northwestern Polytechnical University, Xi’an, 710129 China; 2 School of Computer Science, Harbin Institute of Technology, Harbin, 150001 China

**Keywords:** Gene ontology, Enrichment analysis, Tissue-specific, Web tool

## Abstract

**Background:**

The Gene Ontology (GO) knowledgebase is the world’s largest source of information on the functions of genes. Since the beginning of GO project, various tools have been developed to perform GO enrichment analysis experiments. GO enrichment analysis has become a commonly used method of gene function analysis. Existing GO enrichment analysis tools do not consider tissue-specific information, although this information is very important to current research.

**Results:**

In this paper, we built an easy-to-use web tool called *TS*−*GOEA* that allows users to easily perform experiments based on tissue-specific GO enrichment analysis. *TS*−*GOEA* uses strict threshold statistical method for GO enrichment analysis, and provides statistical tests to improve the reliability of the analysis results. Meanwhile, *TS*−*GOEA* provides tools to compare different experimental results, which is convenient for users to compare the experimental results. To evaluate its performance, we tested the genes associated with platelet disease with *TS*−*GOEA*.

**Conclusions:**

*TS*−*GOEA* is an effective GO analysis tool with unique features. The experimental results show that our method has better performance and provides a useful supplement for the existing GO enrichment analysis tools. *TS*−*GOEA* is available at http://120.77.47.2:5678.

## Background

The Gene Ontology (GO) knowledgebase is the world’s largest source of information on the functions of genes. This knowledge is both human-readable and machine-readable, and is a foundation for computational analysis of large-scale molecular biology and genetics experiments in biomedical research. The goal of the Gene Ontology Consortium is to produce a dynamic, structured, controlled vocabulary that cover several domains of molecular and cellular biology [1]. GO and GO annotations provide a convenient way for biologists to explore the function of gene sets in biological experiments. In detail, GO terms represent a kind of biological knowledge which describes the functions of genes and corresponding gene products [2]. As a unified knowledge base, GO provides three accessible independent ontology, namely biological processes(BP), cellular components(CC) and molecular functions(MF). GO has been widely used in molecular biology and genomics research to describe gene products [1, 3]. In addition, GO provides an ontology annotation system that associates genes or gene products with GO terminology to form a “snapshot” of current biological knowledge. Biologists can design experiments based on GO to verify their biological hypothesis [1, 3–8].

Gene function inference is important in lots of researches [9–12]. The goal of Gene Ontology Enrichment Analysis (GOEA) is to use the annotations of the gene set to find out which GO terms are overrepresented or underrepresented [13, 14]. GOEA has become a common method for functional research of large-scale genome or transcriptome data [15]. Existing GOEA tools can be summarized into two categories, web-based and offline-based application. Offline-based tools require users to download the package and install a local environment, such as BinGO [16], which is not convenient for users to use. At the same time, web-based GOEA tools are very popular with biologists because of its simplicity and convenience, such as DAVID [17], g:profiler [18], GOEAST [15] and GOrilla [19]. However, current GOEA tools do not consider tissue-specific information, and most existing biological experiments do not focus on tissue-specific gene regulation, ignoring their importance in their respective networks [20, 21]. Although all human tissues have a common process, the gene expression patterns of tissues are different, which means that different regulatory procedures control the specificity of the tissue, gene regulation is understood differently in different tissues [21]. Understanding the specific expression and regulation of genes in different tissues is helpful to better understand the genetic relationship and etiology of tissues, as well as to discover new tissue-specific drug targets [22]. Therefore, it is very important to consider tissue-specific genes in current research.

In addition, existing tools simply show the results of enrichment analysis, but they do not show users the relationship between those GO Terms in the results of enrichment analysis. We believe that visualizing the relationship between these GO terms can help us better understand our experimental results.

In order to improve these shortcomings mentioned above, based on Homo sapiens’ GO Annotated data and The Genotype-Tissue Expression [23] data, we constructed an easy-to-use web tool called *TS*−*GOEA*, which allows users to easily conduct experiments based on organization-specific Go enrichment analysis. It uses appropriate statistical methods to determine whether the Go term significantly enriches specific organizations based on a given gene list. Compared with existing tools, it has the following advantages:

- As far as we know, *TS*−*GOEA* is the first tool to provide GO enrichment analysis based on Tissue specificity.
- *TS*−*GOEA* is an easy-to-use Web application that provides an intuitive visual interface that shows the location of specific GO terms in the ontology, as well as the relationships between all enriched Go terms.
- *TS*−*GOEA* can save the results of many experiments, and support the comparison between the results of two groups of different experiments.

## Materials and methods

**Fig. 1:**
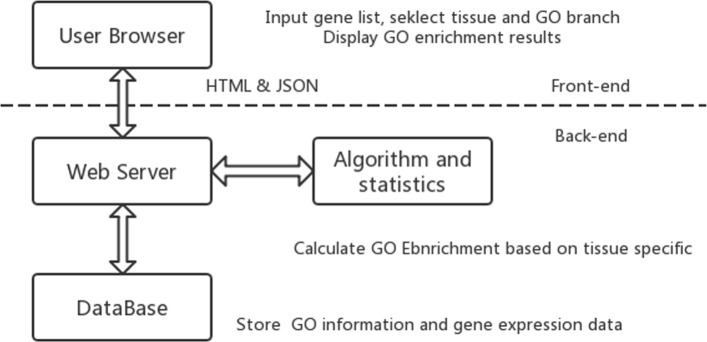
The whole framework of TS-GOEA. The front-end provides a user browser which inputs gene list and displays corresponding GO enrichment results. Calculating GO enrichment based on Tissue specific is finished in the back end of *TS*−*GOEA*

**Fig. 2:**
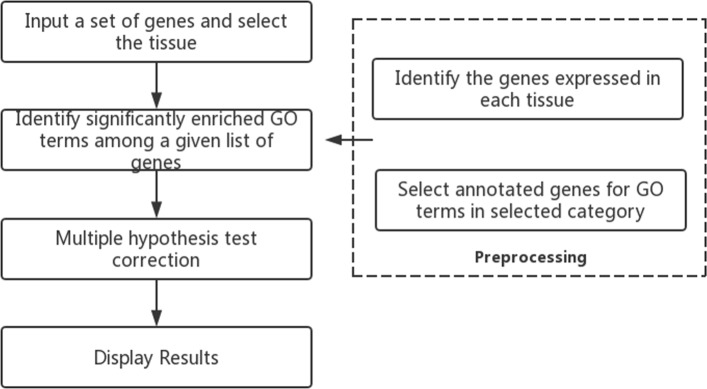
Workflow of tissue-specific GO enrichment analysis

*TS*−*GOEA* is a Web tool with three main layers: data support layer (back-end annotation database); data mining layer (algorithm and statistics); and result presentation layer (interface). The whole framework of *TS*−*GOEA* is shown in Fig. 1 and the workflow of *TS*−*GOEA* is shown in Fig. 2.

### Data resource

The data used by *TS*−*GOEA* comes from the following resources. The GO ontology file is downloaded from the Gene Ontology Project website(http://www.geneontology.org/). All GO term definitions and hierarchical relationships are extracted from the ontology file. The GO annotation file is downloaded and parsed from the Gene Ontology Project website to extract relevant GO terms. Gene expression data was downloaded from the GTEx website(https://gtexportal.org/) and genes for tissue-specific expression were calculated.

### Inputs and outputs format

*TS*−*GOEA* requires the user to enter a list of genes, we currently use as UniPortKB identifier. Besides, *TS*−*GOEA* provides three types of output files:

- HTML table, which describes detailed information of enriched GO terms and corresponding NCBI links.
- Plain text files of GO terms for local processing and analysis.
- Graphical Visualization, showing the hierarchical relationships between all enriched GO Term in the GO category and the hierarchical relationships of each GO term.

### Identify genes expressed on different tissues

Strictly, tissue-specific genes refer to genes whose function and expression are limited to specific tissue or cell types. In many cases, however, the concept of specificity has been extended to tissue selectivity, where gene expression is abundant in one or more tissue/cell types.

The Genotype Tissue Expressions (GTEX) project aims to establish a common resource database and related organization library for studying the relationship between genetic variation and gene expression and other molecular phenotypes in a variety of reference tissues [23, 24]. For ease of study, GTEx dataset provides Transcripts per Million (TPM) value and read counts of genes in different tissues. Select genes that are specifically expressed in tissues based on the following principles [25]:

- In at least 20% samples, TPMs fraction is greater than or equal to 0.1.
- in at least 20% of samples, reads (unnormalized) greater than or equal to 6

#### Hypergeometric test

*TS*−*GOEA* uses hypergeometric testing to calculate possibility. The *p*-value could be calculated as:

1 $$ P(X=x>k)=\sum_{x=k}^{M}\frac{\dbinom{M}{x}\dbinom{N-M}{n-x}} {\dbinom{N}{n}}  $$

Where, *N* is the size of genes specifically expressed in the tissue selected, for a given GO term, there are *M* genes within *N* associated with it, and *n* is the size of genes in the input gene list, *k* is the size of the genes of interaction between *n* and *M* [15]. *TS*−*GOEA* use the Benjamini Hochberg method to adjust the original p value to the error detection rate (FDR) to avoid multiple test problems that may lead to excessive false positive results [26].

### Features of *TS*−*GOEA*

The primary function of *TS*−*GOEA* is to identify statistically enriched GO terms in a given list of genes. As a web-based GO enrichment analysis tool, *TS*−*GOEA* has the following improvements or unique features compared to available tools.

#### Tissue specificity

None of the current GO enrichment analysis tools can take into tissue-specific information account. However, studying the tissue-specific genes is an important step in understanding the progress of life activities and organizational functions. *TS*−*GOEA* performs GO enrichment analysis based on tissue specific information can effectively supplement the shortcomings of current research and better explain the results of biological experiments.

#### Graphical visualization

**Fig. 3:**
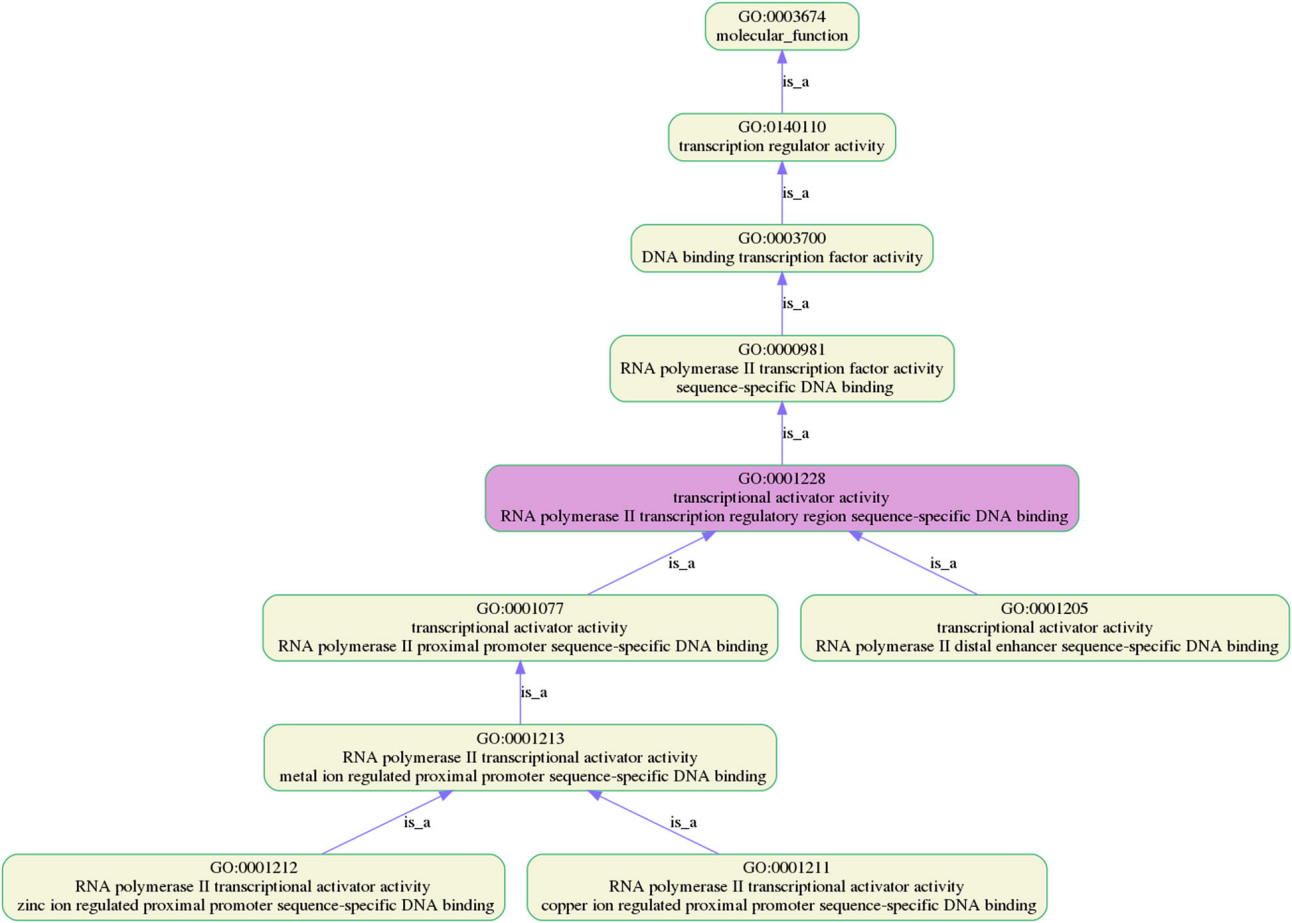
Ancestors and descendants of GO:0001228 in GO

The GO terms in each ontology category are not independent but are located in the same branch, with a hierarchical relationship to each other. Understanding the locational relationships of GO terms may help users better understand their results. For Example, the relative position relationship of GO:0001228 in gene ontology is shown in Fig. 3. With the GO lineage diagram, one can easily understand the enriched GO terms and its hierarchical relationship in GO.

#### Multiple experiments comparison function

A unique feature of *TS*−*GOEA* is to allow comparison of GO terminology enrichment states for different experimental results. Users can upload the results of the GO enrichment analysis provided by *TS*−*GOEA* to the website, or add the results to the comparison page, and compare the similarities and differences between the two experimental results using the Venn diagram.

#### Highly interactive

The application is highly interactive and can generate different diagrams according to user’s selection. For example, in the input interface, users can freely choose interested tissue and a GO category. In the output interface, users can easily download or display their own result. In the result display interface, users can click the GO term list and gene list in the results to view detail information. Users can also compare the results of two enrichment results by adding their job IDs.

We will compare the results of two different GO enrichment analysis at the end of the article to show the advantage of tissue-specific GO enrichment analysis.

## Results and discussion

In this case, tissue-specific genes are defined as a group of genes that express in one or several tissues. Identification of these genes contributes to a better understanding of tissue genetic relationships and pathogenicity [22]. However, due to the complex clinical characteristics and highly heterogeneous genetic background of some diseases, it is difficult to make accurate diagnosis [27, 28]. It is of great significance to reveal the molecular mechanism of biomedicine by using disease genome to performing the tissue-specific GO enrichment analysis, and then continue to excavate the results and analyze the biological process or signal pathway in which genes may be involved.

Platelet disease is a hemorrhagic disease caused by a defect in the quantity or quality of platelets. It is not difficult to understand that compared to other organizations. In the process of exploring the pathogenesis of disease, the study of blood tissue can obtain more accurate results. Therefore, we performed GO enrichment analysis in blood tissue to verify the performance of our tools.

**Fig. 4:**
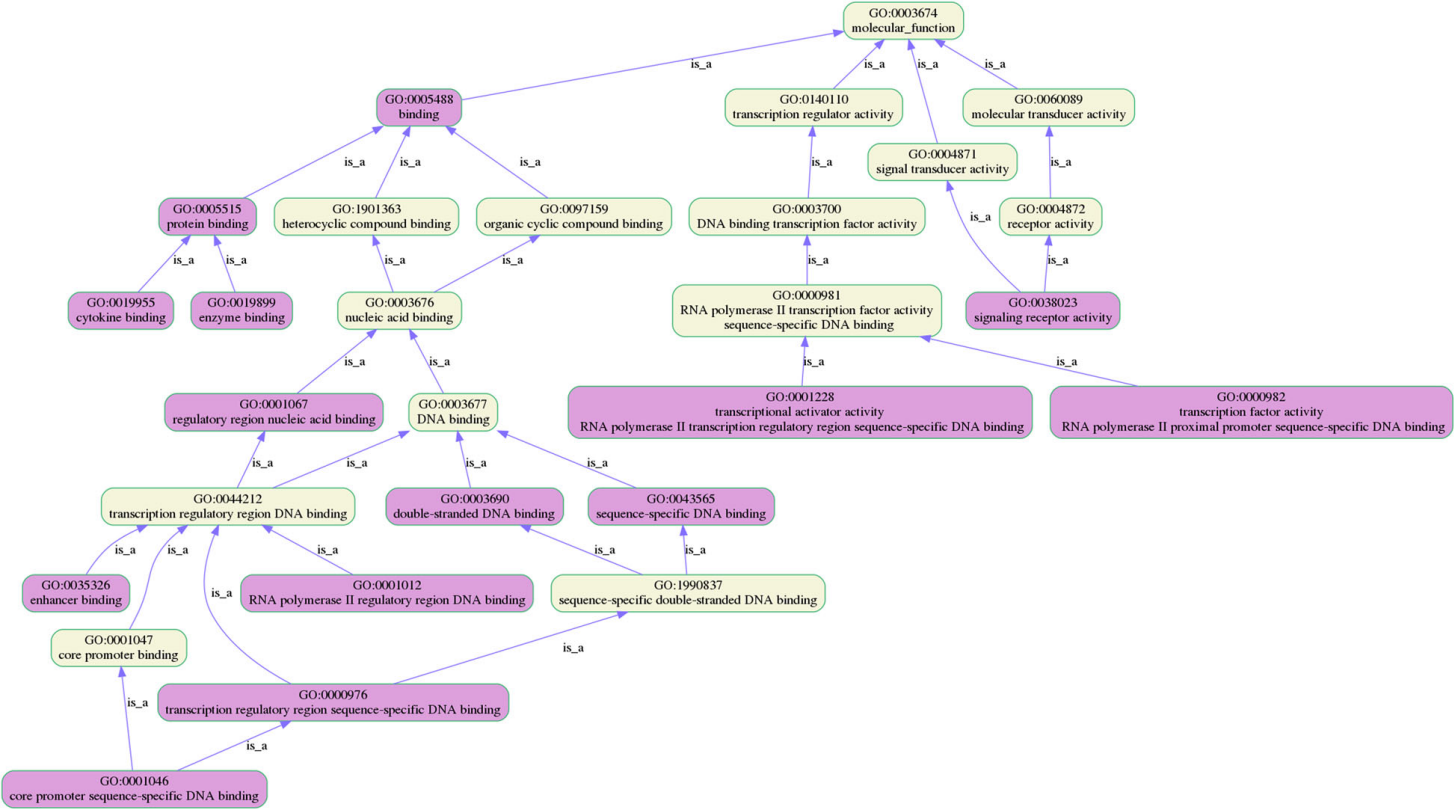
The visualization interface of *TS*−*GOEA*. The experimental result of GO Enrichment analysis in Homo sapiens

**Fig. 5:**
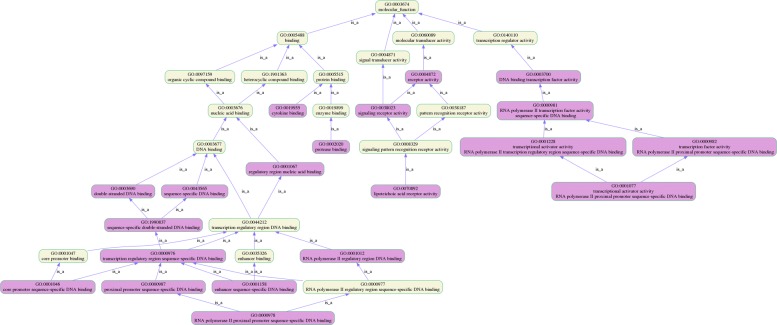
The visualization interface of *TS*−*GOEA*. The experimental result of GO Enrichment analysis in Whole Blood

**Fig. 6:**
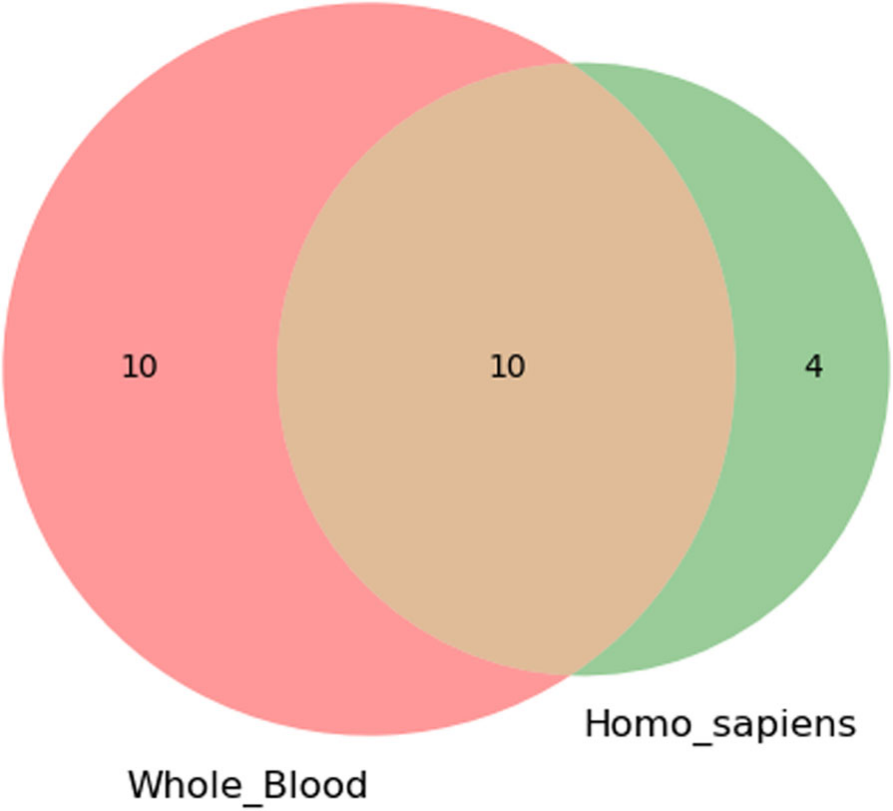
The Venn diagram of two groups of results, which was implemented through the pairwise comparison tool of *TS*−*GOEA*

To test *TS*−*GOEA*, we performed GO enrichment analysis in whole blood tissues and identified a set of GO terms for genes associated with platelet disease. Then, we carried out GO enrichment analysis without using tissue-specific information, and obtained another set of data. We compared and explained the differences between the two groups of experiments. Using the structural relationship of GO, we plot GO lineage images with the results of the two groups of enrichment analysis. Figure 4 shows the result of GO enrichment analysis in Homo sapiens, and Fig. 5 shows the result of GO enrichment analysis in Whole Blood. We compare the two groups of results, as shown in Fig. 6.

By comparing the results of the two sets of experiments, we can find that the GO enrichment analysis based on whole blood tissue produces more accurate and effective results. More specifically, in order to facilitate comparison, we list the GO terms for these differences in the Table 1. Most of these GO terms listed are related to the activity of proteases and DNA binding processes, and are helpful to mediate the transcription process. We enumerate the genes annotated by those GO Terms and search these genes on eDGAR [29]. We found that these genes affect the formation and function of related proteases in the blood, and their abnormal expression can lead to some blood-related diseases, including Platelet disease, which proves the effectiveness of our tools. The results showed that tissue-specific GO enrichment analysis could show information at a more specific level. Therefore, we believe that our tools can help biologists complement and improve the process of biological experiments, understand their results from a functional point of view, and explore the potential molecular mechanisms behind biological processes [15].

**Table 1:** GO TERMS ENRICHED IN WHOLE BLOOD and related disease genes

GO term	GO function	Disease-related gene
GO:0000978	RNA polymerase II proximal promoter sequence-specific DNA binding	GATA1,HOXD13CEBPA,FLI1,NR4A3,CEBPA
GO:0000981	RNA polymerase II transcription factor activity, sequence-specific DNA binding	FLI1,CEBPA,GATA1,GATA2,HOXD13,NR4A3
GO:0000987	proximal promoter sequence-specific DNA binding	CEBPA,GATA1,HOXD13,NR4A3
GO:0001077	transcriptional activator activity, RNA polymerase II proximal promoter sequence-specific DNA binding	GATA1,GATA2,HOXD13,CEBPA,FLI1,NR4A3,CEBPA
GO:0001158	enhancer sequence-specific DNA binding	GATA1,GATA2,HOXD13
GO:0002020	protease binding	VWF,ITGB,ELANE,NR4A3
GO:0003700	obsolete negative regulation of diuresis	GATA1,GATA2,HOXD13,CEBPA,FLI1,CEBPA
GO:0004872	receptor activity	TBXA2R,CD36,F2,GP1BB,MPL,NR4A3
GO:0070892	lipoteichoic acid receptor activity	CD36
GO:1990837	sequence-specific double-stranded DNA binding	CEBPA,GATA1,GATA2,HOXD13,NR4A3

## Conclusion

Since the beginning of the GO project, GO enrichment analysis has become a widely used method in the functional study of large-scale genome or transcriptome data. Various tools have been developed to support the exploration and search of the go database. Specifically, various tools are currently available to perform GO enrichment analysis. However, existing tools ignore tissue-specific information, which may bias the results of biological experiments.

In this article, we developed a Web application that allows users to perform organization-specific GO rich analysis experiments, and we also visualize our results to facilitate users to view the relationships between GO terms. We also provide tools to compare different experimental results, so users can find similarities and differences between different experiments and mine deeper relationships. In a word, *TS*−*GOEA* is an easy-to-use Web application that fills the gap in the field of tissue-specific GO enrichment analysis and can effectively supplement the conclusions of some current biological experiments.

## Abbreviations

BP: Biological processes
CC: Cellular components
FDR: False discovery rate
GO: Gene ontology
GOEA: Gene ontology enrichment analysis
GTEx: The genotype-tissue expression
MF: Molecular functions
TPM: Transcripts per Million
